# Determinants of cigarette smoking and smoking intensity among adult males in Ghana

**DOI:** 10.1186/s12889-018-5872-0

**Published:** 2018-07-31

**Authors:** Edward Nketiah-Amponsah, Gloria Afful-Mensah, Samuel Ampaw

**Affiliations:** 1 Department of Economics, University of Ghana, Legon (Accra), Ghana; 2 Department of Economics, Management and Quantitative Methods, University of Milan, Milan, Italy

**Keywords:** Adult males, Cigarette smoking, Smoking intensity, Two-part model, Logit regression, Negative binomial, Ghana

## Abstract

**Background:**

In spite of the adverse health and financial implications of smoking, it still remains one of the leading causes of preventable diseases and deaths in the world. Key to discouraging the habit of smoking is knowledge of the drivers of smoking. In Ghana, though smoking behaviours are relatively more associated with adult males than youth and adolescents, studies on smoking behaviours of adult males are scant. This study, therefore, investigates the determinants of cigarette smoking and smoking intensity among adult males in Ghana.

**Methods:**

Data were obtained from the most recent Ghana Demographic and Health Survey (DHS) conducted in 2014. Based on the 2014 GDHS, a negative binomial-logit hurdle model was estimated to explore the socioeconomic and demographic characteristics associated with cigarette consumption and smoking intensity among adult males in Ghana. To ensure robustness, separate estimations were performed for the respective logit and negative binomial models used in the two-part model.

**Results:**

We find that men in lower socioeconomic category (poor and low education) have a higher likelihood to smoke. Also, age proved significant in explaining smoking behaviors in Ghana. Moreover, religion and region of residence are reported to affect cigarette consumption decision. Furthermore, we find that among the men who smoke, those between the ages of 44 and 60 years and have attained approximately primary education have a higher likelihood to smoke greater quantities of cigarette daily. Also, the smokers who reside in the Upper East and Upper West regions are reported to smoke more intensely than their counterparts in the Greater Accra region.

**Conclusion:**

Since smoking remains one of the major causes of diseases and deaths the world over, the current study provides recent empirical evidence based on a nationally representative sample for public health policies geared towards smoking reduction and ultimately cessation. This study suggests that public policies that promote higher educational attainment and improved incomes (wealth) are crucial in smoking reduction and cessation in Ghana.

## Background

Although studies have shown that nicotine increases the level of dopamine in the brain, resulting in feelings of pleasure and well-being, evidence abounds on the health implications of smoking. Smoking remains one of the major causes of diseases and deaths the world over [1–4]. Smoking and nicotine engender disease conditions such as asthma and wheezing, cardiovascular diseases and cancer and in particular, pregnant women who smoke predispose their unborn babies to respiratory diseases [5, 6]. Also, apart from the harmful effect of smoking on one’s own health and behaviour, smoking causes health problems for others through secondhand smoke [7, 8]. The WHO estimates that a little over half a million people die every year because of secondhand smoking [7]. In spite of the adverse health and financial implications of smoking, it still remains one of the leading causes of preventable diseases and deaths in the world. For instance, in Australia, smoking accounts for over 15,000 deaths each year, outweighing the total deaths from many other causes [9]. In the 1990s, approximately, a billion people smoked daily in the world, of which about 47% were adult men and about 12% adult women [10]. To minimize the health hazards associated with smoking, the WHO ratified the Framework Convention on Tobacco Control in 2003, and many countries have since implemented a variety of smoking control policies.

In Ghana, the prevalence of smoking is below 10% as evident by the findings in the various surveys carried out by the Ghana Demographic and Health Survey revealing smoking to be relatively predominant among men in Ghana. Estimates by the World Bank’s World Development Indicators show a consistent decline in the proportion of male smokers in Ghana from 10.8% in 2000 to 7.7% in 2016.^1^ Similarly, the proportion of female smokers in Ghana declined from 0.9% in 2000 to 0.3% in 2016 [11]. Nonetheless, any level of smoking is a matter of public health concern because of the negative externalities (i.e. the effect of secondhand smoke) associated with the act.

In terms of empirical literature, a plethora of studies have been conducted on determinants of cigarette smoking in both developed and developing countries [12–18]. However, there is paucity of studies in this area of public health research in Ghana. Yet, the existing studies are limited in scope [1, 14].^2^ In Ghana, tobacco control measures are specified in Article 58 to 68 of the Public Health Act 2012 (Act 851). This part of the Act clearly provides information such as the prohibition of smoking in public places, advertisement of tobacco and tobacco products, packaging and labelling, minimum age restrictions and sale of tobacco products. Though the Act shows Ghana’s commitment to the WHO’s Framework Convention, unfortunately, not much has been done regarding the enforcement of these policies. Also, not much is seen in the various media platforms in the form of educational campaigns and advertisement on the health effects of smoking. In the literature, there is a lacuna with regard to the empirical evidence on the driving factors of smoking in Ghana probably due to the relatively low prevalence rate of smoking in the country. This study is motivated by three main reasons. First, it provides some empirical insight into the factors influencing the “few” smokers’ decision to smoke given that Africa is gradually becoming a potential market for the tobacco industry to explore. Second, given that smoking is a habit that can be acquired through interactions; the epidemic spread of smoking may be inevitable. Therefore, for public health policy purposes, there is certainly the need to find the driving factors for smoking in order to avoid uncontrollable levels in the future. This may even help in the cessation of smoking. Finally, the health implication of smoking on passive smokers is as bad as that on the primary smoker even though the percentage of primary smokers may be low in Ghana.

A number of factors have been revealed in the empirical literature elsewhere to influence a person’s decision to smoke. In a study by Nguyen [17], factors such as gender, age (middle-aged men), people of low education and working people were found to be more likely to smoke in Vietnam. Also, being a widow was found to increase the probability of smoking. Generally, factors influencing smoking behavior encompassed intrapersonal characteristics and economic factors. Other studies had investigated the impacts of smoking control policies on smoking choice [19]. After controlling for important covariates including age, gender and socio-economic status, Grenard et al. [20] identified interpersonal influences (parental monitoring, good friend smoking, and peer smoking), attitudinal/cultural influences (school academic ranking, initial liking of smoking and the meaning of smoking) and intrapersonal influences (susceptibility to smoking, and low-self-confidence to quit smoking) as significant risk factors for smoking.

In a related study, Rudatsikira et al. [21] attempted to estimate the prevalence of smoking and its association among school-going adolescents in Addis Ababa, Ethiopia using data from the Global Youth Tobacco Survey (GYTS), 2003. Using bivariate and multivariate logistic regression analyses, it was found that having smoking friends was strongly associated with smoking after controlling for age, gender, parental smoking status, and perception of risks of smoking [21]. They further found that gender (male) and having one or both parents as smokers were significantly associated with smoking. The perception that smoking is harmful was found to be significant and negatively associated with being a smoker. This finding somehow suggests the efficacy of knowledge acquired via educational campaigns on the adverse health implications of smoking. Thus, knowledge of the harmful impact of smoking is key in combating the menace.

Similarly, Turrell et al. [22] examined the influence of neighbourhood disadvantage on smoking cessation among the residents of Brisbane, Australia. Their study used a multilevel logistic regression and Markov chain Monte Carlo simulation in their analysis. After controlling for individual-level socioeconomic factors, they found that the probability of quitting smoking between 2007 and 2009 was lower for residents of disadvantaged neighbourhoods than their counterparts in more advantaged neighbourhoods. This study, therefore, brought to light the need to improve socio-economic conditions in the deprived areas in societies in our collective effort to curb smoking.

Furthermore, Ross et al. [23] observed how cigarette prices, smoking restrictions, and limits on youth access to tobacco could affect smoking uptake among adolescents. Using a generalized ordered logit model, the results indicated that higher cigarette prices are associated with lower smoking uptake and that higher prices have an increasing impact as an individual faces larger risk of becoming an established smoker. Their study also revealed that compliance with youth access laws can slow down smoking uptake progress among youth.

It is well-established that more males than females smoke [24]. In particular, the study revealed that 47% of all men while only 11% of all women smoke. However, the proportion of females who smoke in the advanced countries is gradually getting closer to that of their male counterparts. The gender differences in smoking have necessitated the inclusion of gender in most studies. In an attempt to explain this gender differences, a study [25], has identified three main reasons for gender differences in smoking behaviour: (i) the general characteristics of the traditional sex roles lead to social pressure against female smoking, (ii) traditional sex role norms cause differences in personal characteristics leading to more or less acceptance of smoking (e.g. rebelliousness among males is more accepted than among women and this causes higher smoking rates) and (iii) sex roles influence the assessment of the costs and utility associated with smoking.

Understanding factors influencing the smoking status of adult men is of interest to policymakers as well as researchers because the health burden and associated cost of smoking go beyond the smoker himself/herself. Consequently, this paper examines the phenomenon using the most recent Ghana Demographic and Health Survey (GDHS) conducted in 2014. Since smoking or the use of tobacco products among females is almost negligible in Ghana, the empirical analysis carried out in this study was done using only the male sample (i.e. men in their reproductive stage).^3^ However, we also provide some descriptive statistics with regard to female smokers in the results section. Specifically, we provide some background characteristics of female smokers. Knowing the characteristics of female smokers may serve as a first step in terms of policy design and perhaps inform some policy directions on how to maintain low level or a declining level of female smokers.

The rest of paper is organized as follows. The second and third sections present the method of analysis and results of the study respectively while section four discusses the results. Section five concludes the paper with a recap of the key findings and their policy implications.

## Methods

### Data

The study utilizes the most recent data on Ghana from the global Demographic and Health Surveys (DHSs). These surveys are countrywide population and health surveys. Six surveys have been conducted in Ghana since 1988 by the Ghana Statistical Service (GSS), Ghana Health Service (GHS), and ICF International. The 2014 Ghana Demographic and Health Survey (GDHS) followed a two-stage sampling strategy. The first stage was the selection stage. Here, sample points (clusters) comprising enumeration areas (EAs) were randomly selected. The second stage involved the systematic sampling of households. Because the administrative regions approximately had equal sample sizes, the 2014 GDHS was not self-weighted at the national level [26]. The implementation of the survey encountered sampling and non-sampling errors [26].

Altogether, 11,835 households were interviewed. Although 4609 eligible men and 9656 eligible women from 15 years to 59 years and 15 years to 49 years respectively were identified for the interview, 4388 men and 9396 women were successfully interviewed. These yielded response rates of about 95 and 97% respectively [26]. The study used data from the men’s questionnaire. Of the total men interviewed, 4385 observations were used for the study as these were the observations with complete information for all the variables of interest.

### Outcome variables

The 2014 GDHS collected information on cigarette consumption status as well as the number of cigarettes smoked by respondent smokers. In the survey, the male respondents were asked whether or not they smoke cigarette. “Yes” was selected for respondents who smoke cigarette and “no” otherwise. This response was used in exploring the socioeconomic and demographic determinants of cigarette smoking among the male sample.

A further question on the number of cigarette sticks smoked in the last 24 h was posed to the respondents who smoke. The study adopted the response to this question to examine the determinants of smoking intensity among adult males in Ghana. Description and measurement of the outcome variables used in the study are presented in Table 1.

**Table 1:** Description and Measurements of Outcome Variables used in the Estimations

Outcome, Variables	Description	Measurement
Smokes	Cigarette smoking status	1 = smokes, otherwise = 0
Smoking intensity	Smoking frequency	Number of cigarette sticks smoked in last 24 h

### Explanatory variables

Other socioeconomic and demographic characteristics (including inter alia, age, educational attainment, health insurance status, employment status, religious affiliation, region of residence, and frequency of use of radio, TV, and newspapers) are contained in the dataset obtained from the 2014 GDHS. Description and measurement of the selected explanatory variables are presented in Table 2.

**Table 2:** Description and Measurements of Explanatory Variables used in the Estimations

Explanatory Variables	Description	Measurement
15–29 years	Age category	1 = 15–29 years; otherwise = 0
30–44 years	Age category	1 = 30–44 years; otherwise = 0
45–59 years *(ref.)*	Age category	1 = 45–59 years; otherwise = 0
Education	Years of education	Number of years spent in school
Education squared	Years of education squared	square of number of years spent in school
Old*educated	Age and education interacted	1 if years of education is greater than 0 and 44 years < age < 60 years, otherwise 0
Use of TV	Utilization of television	1 = sometimes, 0 = never
Lower*(ref.)*	Wealth category	1 = lower wealth category, otherwise = 0
Middle	Wealth category	1 = average wealth category, otherwise = 0
Higher	Wealth category	1 = higher wealth category, otherwise = 0
Other religion *(ref.)*	Religious affiliation	1 = other religions aside from Christianity and Islam, otherwise = 0
Christianity	Religious affiliation	1 = Christianity, otherwise = 0
Muslim	Religious affiliation	1 = Islam, otherwise = 0
Western	Region of residence	1 = Western, otherwise = 0
Central	Region of residence	1 = Central, otherwise = 0
Greater Accra *(ref.)*	Region of residence	1 = Greater Accra, otherwise = 0
Volta	Region of residence	1 = Volta, otherwise = 0
Eastern	Region of residence	1 = Eastern, otherwise = 0
Ashanti	Region of residence	1 = Ashanti, otherwise = 0
Brong Ahafo	Region of residence	1 = Brong Ahafo, otherwise = 0
Northern	Region of residence	1 = Northern, otherwise = 0
Upper East	Region of residence	1 = Upper East, otherwise = 0
Upper West	Region of residence	1 = Upper West, otherwise = 0

*Connotes interaction between the two variables

### Statistical analysis

A negative binomial-logit hurdle model was used to investigate the determinants of cigarette smoking and smoking intensity among adult males in Ghana. According to [27], the hurdle model is a two-part model used in modeling count data. [27, 28] further report that a hurdle model includes a two-stage modeling process in which the first stage is a binary model and the second stage is a truncated model. In the first stage of the hurdle model applied in this study, people decide whether to smoke or not. Since this involves a binary decision, a logit model is employed. In the second stage of the model, conditional on smoking, the smokers decide the number of cigarette sticks to smoke. A negative binomial model is adopted in the second stage to model the over-dispersed^4^ count data. This is motivated by the suggestion that the assumption of equidispersion^5^ of the Poisson model is unreal [29]. However, the choice of the negative binomial model over the Poisson model was tested empirically. For robustness, the respective empirical models which were estimated jointly in the two-part model and separately are specified as below.

Logit Model:

1 $$ {\mathrm{Smokes}}_{\mathrm{i}}={\beta}_0+{\beta}_1\mathrm{Age}\kern.3em {\mathrm{category}}_{\mathrm{i}}+{\beta}_2{\mathrm{Education}}_{\mathrm{i}}+{\beta}_3\mathrm{Education}\kern.3em {\mathrm{squared}}_{\mathrm{i}}+{\beta}_4{\mathrm{Age}}_{\mathrm{i}}\ast {\mathrm{Education}}_{\mathrm{i}}+{\beta}_5\mathrm{TV}\kern.3em {\mathrm{use}}_{\mathrm{i}}+{\beta}_6\mathrm{Wealth}\kern.3em {\mathrm{category}}_{\mathrm{i}}+{\beta}_7{\mathrm{Religion}}_{\mathrm{i}}+{\beta}_8{\mathrm{Region}}_{\mathrm{i}}+{\upvarepsilon}_{\mathrm{i}} $$

Negative Binomial Model:

2 $$ \mathrm{Smoking}\kern.3em {\mathrm{i}\mathrm{ntensity}}_{\mathrm{i}}={\beta}_0+{\beta}_1\mathrm{Age}\kern.3em {\mathrm{category}}_{\mathrm{i}}+{\beta}_2{\mathrm{Education}}_{\mathrm{i}}+{\beta}_3\mathrm{Education}\kern.3em {\mathrm{squared}}_{\mathrm{i}}+{\beta}_4\mathrm{Ag}{\mathrm{e}}_{\mathrm{i}}\ast {\mathrm{Education}}_{\mathrm{i}}+{\beta}_5\mathrm{TV}\kern.3em {\mathrm{use}}_{\mathrm{i}}+{\beta}_6\mathrm{Wealth}\kern.3em {\mathrm{category}}_{\mathrm{i}}+{\beta}_7{\mathrm{Religion}}_i+{\beta}_8{\mathrm{Region}}_{\mathrm{i}}+{\upvarepsilon}_{\mathrm{i}} $$

All statistical analyses were done using Stata version 13. In estimating the separate logit model, the survey design was taken into account. Thus, the estimated results are nationally representative. The model fits for the estimated models were evaluated and reported. Apart from the estimation of the separate logit model which used linearized standard errors in estimating the significance of the coefficients, robust standard errors were used in the other models.

## Results

### Prevalence of smoking

Table 3 presents a trend analysis of cigarette smoking among men and women in Ghana. This analysis was done between years 2003 and 2014 because the last three rounds of the Ghana Demographic and Health Surveys were conducted within this period. To yield nationally representative estimates, sample weights were applied in our estimation of the descriptive statistics presented in Table 3. The results suggest that more men than women in Ghana smoke cigarette. Prevalence almost doubled for women between 2003 and 2008 and it roughly halved again by 2014. On the other hand, the prevalence among males declined consistently throughout. For the respective samples to be nationally representative, the survey design was accounted for in the estimations. Results from the 2014 dataset show that though 253 men out of the total 4388 men sampled^6^ smoke cigarette, only 5 women out of the 9396 women interviewed^7^ reported that they smoke cigarette.

**Table 3:** Prevalence of Cigarette Smoking By Gender, 2003–2014

Smokes cigarette	2003 GDHS		2008 GDHS		2014 GDHS	
	Male	Female	Male	Female	Male	Female
No	90.95	99.91	92.69	99.83	95.22	99.93
Yes	9.05	0.09	7.31	0.17	4.78	0.07

Column percentages reported. *Source: Computed by authors from the 2003, 2008 & 2014 GDHS*

Generally, from Table 4, smoking among females is typically recorded among the poorest (lowest) wealth quintile and those with no formal education. The Northern region recorded the highest proportion (80.65%) of female smokers. Compared to their counterparts in the urban areas, majority (87.10%) of female smokers reside in the rural areas. In Ghana, poverty is endemic in rural areas and thus the high prevalence of smoking in rural areas may be largely attributable to their poor economic conditions. The stress associated with lack of employment opportunities and low incomes for those employed mainly in the agricultural sector may plunge some women into smoking as one may see smoking as a temporary relief for economic hardships..

**Table 4:** Female smokers by selected background characteristics

Variable	Frequency	Percentage
*Age (in group)*		
15–19	0	0.00
20–24	2	6.45
25–29	2	6.45
30–34	5	16.13
35–39	8	25.81
40–44	5	16.13
45–49	9	29.03
*Place of residence*		
Urban	4	12.90
Rural	27	87.10
*Region of residence*		
Western	0	0.00
Central	1	3.23
Greater Accra	0	0.00
Volta	0	0.00
Eastern	1	3.23
Ashanti	0	0.00
Brong Ahafo	2	6.45
Northern	25	80.65
Upper East	1	3.23
Upper West	1	3.23
*Wealth quintile*		
Poorest	28	90.32
Poorer	2	6.45
Middle	0	0.00
Richer	1	3.23
Richest	0	0.00
*Level of formal education*		
No education	27	87.10
Basic	1	3.23
Secondary	3	9.68
Higher	0	0.00
Observations	31	

*Source: Computed by authors from GDHS 2014*

### Summary statistics of variables

Table 5 shows that approximately 5% of the sampled men smoke cigarette. Among the smokers, an average of about 6 sticks is smoked daily. Majority of the sampled men, 46.3%, are in the 15–29 years category. Averagely, the sampled men spent about 9 years in school. This is equivalent to the completion of Junior Secondary education. About 16% of the older men (45–59 years) have ever attended school. Also, a majority of the men, 83.2% watch television. Almost half of the sampled men, 46.1%, are in the higher wealth category. Besides, roughly 73% of the men are Christians. Lastly, about 86% of the sampled men live in the southern regions of Ghana.

**Table 5:** Summary Statistics of Variables used in the Estimations

Variables	Mean	SE
Smokes	0.048	0.004
Smoking intensity^+^	5.731	–
15–29 years	0.463	0.010
30–44 years	0.338	0.010
45–59 years *(ref.)*	0.199	0.007
Education^+^	9.069	0.135
Education squared	–	–
Old*educated	0.161	0.007
Use of TV	0.832	0.012
Lower*(ref.)*	0.349	0.015
Middle	0.190	0.011
Higher	0.461	0.016
Other religion *(ref.)*	0.098	0.007
Christianity	0.726	0.013
Muslim	0.176	0.014
Western	0.114	0.009
Central	0.096	0.016
Greater Accra *(ref)*	0.210	0.012
Volta	0.077	0.006
Eastern	0.098	0.006
Ashanti	0.180	0.011
Brong Ahafo	0.083	0.005
Northern	0.081	0.009
Upper East	0.038	0.004
Upper West	0.023	0.002

All but *smoking intensity* and *education* are in proportions. SE denotes linearized standard error. Reference category denoted by *ref*. Missing standard error for the smoking intensity because of stratum with single sampling unit. *Source: Computed by authors from GDHS 2014*

### Bivariate analysis (explanatory variables by smoking status)

Table 6 presents a bivariate analysis of the selected explanatory variables by smoking status. Majority of the men who smoke, 47.5%, are in the 45–59 years category while 32.1 and 20.3% of them are in the 30–44 years and 45–59 years categories respectively. On average, the sampled men who do not smoke spend about 4 more years in school than their counterparts who smoke. Furthermore, a lower percentage of the men who smoke, 27.3%, are older (45–59 years) and have ever attended school. Besides, majority of the men who smoke, 63.35%, sometimes watch television. While majority of the men who do not smoke, 47.6%, are in the higher wealth category, most of the men who smoke are in the lower wealth category. Also, more of the sampled men are Christians. The results presented in Table 5 further show that while majority of the men who do not smoke, 21.4%, live in the Greater Accra region, most of the men who smoke,16.8%, reside in the Northern region of Ghana.

**Table 6:** Bivariate Analysis of Explanatory Variables by Smoking Status

Explanatory	Cigarette Smoking			
Variables	No	Yes	F-statistic	*P*-value
*Age Category*			44.195	0.0000
15–29 years	0.476	0.203		
30–44 years	0.339	0.321		
45–59 years	0.185	0.475		
*Education*	9.278	4.894	8.579	0.0000
Age*Education (Other)			12.032	0.0006
Old*educated	0.156	0.273		
*Use of TV (Never)*			51.646	0.0000
Sometimes	0.843	0.624		
*Wealth category*			26.844	0.0000
Lower	0.335	0.635		
Middle	0.189	0.211		
Higher	0.476	0.154		
*Religious affiliation*			60.165	0.0000
Other religion	0.087	0.324		
Christianity	0.744	0.359		
Muslim	0.169	0.317		
*Religion*			6.054	0.0000
Western	0.116	0.072		
Central	0.097	0.078		
Greater Accra	0.214	0.126		
Volta	0.077	0.071		
Eastern	0.100	0.056		
Ashanti	0.181	0.168		
Brong Ahafo	0.082	0.100		
Northern	0.076	0.191		
Upper East	0.035	0.095		
Upper West	0.022	0.044		

Column proportions reported. Survey design accounted for in estimations. *** *p* < 0.01, ** *p* < 0.05, * *p* < 0.1. *Source: Computed by authors from GDHS 2014*

The statistical significance of the F-statistic at 1% level of significance (*p*-value< 0.01) for all the selected variables show that cigarette smoking decision among adult males in Ghana are highly associated with the respective variables. Hence, all the variables used in the bivariate analysis were used in the multivariate analysis to explore the determinants of cigarette smoking among Ghanaian adult males.

### Multivariate analysis

Table 7 presents the results from the two-part model (labels 1 and 2) as well as that from the separate estimations of the logit (label 3) and negative binomial (label 4) models. Eight (8) variables were used to explore the determinants of cigarette smoking and smoking intensity among adult males in Ghana. The statistical significance of the Wald chi2 test in the two-part model and the negative binomial model and that of the F-statistic of the logit model indicate the robustness of the respective models in explaining the determinants of cigarette smoking and smoking intensity. Besides, the Logit model’s F-adjusted mean residual test of goodness-of-fit is insignificant, an indication that the model fits the data well. Also, the significance of alpha in the negative binomial model justifies the choice of a negative binomial model over Poisson model. Hence, by using negative binomial model, the study corrects for potential unobserved heterogeneity. Lastly, the lower value of the hurdle model’s AIC indicates a better fit of the model. But for the counter-intuitive findings for the religion variables which are adequately justified in the discussion, the signs on the significant variables in the logit model reconcile with those in the negative binomial model.

**Table 7:** Determinants (Coefficients) of Cigarette Consumption and Smoking Intensity

Explanatory	Two-Part Model^a^		Logit Model	Negative Binomial Model
Variables	(1)^b^	(2)^c^	(3)	(4)
*Age Category (45–59 years)*				
15–29 years	−14.002(1.935) ***	−0.414(0.180) **	−1.232(0.299) ***	−0.416**
30–44 years	−12.939(1.417) ***	−0.069(0.139)	−0.601(0.238) **	−0.048(0.128)
*Education*				
Years of education	−0.335(0.327)	−0.054(0.037)	−0.158(0.063) **	−0.066(0.036) *
Years of education squared	0.029(0.019)	0.005(0.003) *	0.004(0.005)	0.006(0.003) **
Age*Education (Others)				
Old*educated	1.189(1.671)	0.307(0.184) *	0.499(0.341)	0.342(0.170) **
*Use of TV (Never)*				
Sometimes	−0.515(1.344)	0.077(0.128)	−0.236(0.192)	0.053(0.113)
*Wealth category (Lower)*				
Middle	−0.081(0.854)	0.209(0.163)	0.114(0.240)	0.172(0.146)
Higher	−0.023(1.280)	0.162(0.205)	−1.034(0.332) ***	0.183(0.190)
*Religious affiliation (Others)*				
Christian	−0.275(1.279)	0.051(0.137)	−1.265(0.241) ***	0.045(0.122)
Muslim	−0.762(1.228)	0.614(0.141) ***	−0.507(0.252) **	0.548(0.126) ***
*Region (Greater Accra)*				
Western	−0.512(1.001)	−0.699(0.336) **	− 0.804(0.521)	−0.570(0.277) **
Central	14.608(1.670) ***	−0.278(0.350)	−0.533(0.499)	− 0.109(0.281)
Volta	14.528(1.700) ***	−0.082(0.362)	−0.659(0.525)	0.045(0.304)
Eastern	0.088(1.179)	−0.582(0.328) *	−1.002(0.541) *	−0.432(0.268)
Ashanti	14.432(0.841) ***	−0.185(0.310)	−0.060(0.471)	− 0.030(0.252)
Brong Ahafo	0.673(1.165)	−0.066(0.315)	−0.407(0.512)	0.052(0.243)
Northern	14.880(1.121) ***	0.065(0.329)	−0.478(0.529)	0.194(0.267)
Upper East	1.077(0.942)	0.668(0.305) **	−0.280(0.500)	0.730(0.243) ***
Upper West	15.257(0.975) ***	0.439(0.315)	−0.344(0.551)	0.525(0.255) **
Constant	16.454(1.299) ***	1.322(0.315) ***	0.049(0.467)	1.302(0.250) ***
Observations	252	252	4385	252
Wald chi2^+^	3847.69 (0.000) ***		–	156.93 (0.000) ***
F-statistic^+^	–		12.89 (0.000) ***	–
F-adjusted mean residual test^+^	–		0.506 (0. 870)	–
Pseudo-R2	–		–	0.0713
Lnalpha	−0.995(0.219) ***		–	0.307(0.056) ***
Alpha	–		–	−1.180(0.182) ***
AIC statistic	5.319		–	–

Robust standard errors in parentheses. *P*-values in parentheses for those marked +. Estimation of the separate logit model accounted for the survey design and hence applied linearized standard errors. *** *p* < 0.01, ** *p* < 0.05, * *p* < 0.1

*Source: Computed by authors from GDHS 2014*

^a^ Negative Binomial-Logit Hurdle Model

^b^ Logit Model

^c^ Negative Binomial Model

The results indicate that men in lower socioeconomic and demographic categories (aged, poor, and low education) have a higher likelihood to smoke. This is because compared to the older men (45–59 years), the younger men (15–29 years and 30–44 years) are found to have a lower likelihood to smoke. Additionally, the men in higher wealth category are reported to be less likely to smoke than their counterparts in the lower wealth category. Years of education is also found to be negatively associated with the likelihood of smoking. The above findings are consistent with existing literature. Compared to men who are affiliated with other religious groups, those affiliated with Christianity or Islam are associated with having a lower smoking probability. The study further finds region of residence to be an important predictor of the likelihood of cigarette smoking among adult males in Ghana. Compared to men who live in the Greater Accra region, those in the Central, Volta, Ashanti, Northern, and Upper West regions are found to be more likely to smoke cigarette.

Regarding the determinants of cigarette smoking intensity, the study finds that younger men who smoke cigarette (15–29 years) have a lower likelihood to smoke greater quantities of cigarette than their counterparts in the older age category (45–59 years). Additionally, among the men who smoke, those between the ages of 44 and 60 years and have attained approximately primary education^8^ have a higher likelihood to smoke greater quantities of cigarette. Though Muslims are found to be less likely to smoke, those who smoke are revealed to have a higher likelihood to smoke more often than their counterparts who are affiliated with other religions apart from Christianity. The study further finds that compared to smokers who live in the Greater Accra region, their counterparts who reside in the Upper East and Upper West regions are reported to have a higher likelihood to smoke greater quantities of cigarette. However, the smokers who reside in the Western region are found to have a lower probability of smoking intensely.

## Discussion

### Determinants of cigarette consumption and smoking intensity

Older Ghanaian men are found to be more likely to smoke and also smoke greater quantities of cigarette than their younger counterparts. This finding is consistent with that of [11, 30, 31] who reported similar findings in Ethiopia, Ghana, and Nepal respectively. Despite the fact that health stock depreciates with age [32], the current study found that Ghanaian male adults have a higher probability to engage in smoking as they age. Habits formed are difficult to break. The greater likelihood of smoking among males may be attributable to their addiction to smoking since their youthful years. Besides, male adults often have huge financial responsibilities to the extent that some men are unable to meet their basic needs of housing, education, and health and this is usually a recipe for smoking which may be seen as an escape route to stress and anxiety [33].

Education creates the needed awareness about the debilitating health impact of cigarette smoking. Thus, a policy to ensure universal access to education is proven to be useful in controlling the smoking of cigarettes by Ghanaian male adults. In this regard, the free senior high school (SHS) campaign promise made by the ruling government of Ghana will possibly aid in curbing cigarette smoking among male adults in the future. This is based on the fact that years of education is negatively associated with the likelihood of smoking and also, smoking intensity. Nguyen [17] and Khana [31] made similar findings in Vietnam and Nepal respectively. Because educated individuals are fully aware of the numerous health risks and addictive prowess associated with the consumption of cigarettes, they have a higher likelihood to desist from smoking. Further, among the men who smoke, those who spend more than about 6 years in school are found to have a higher probability of smoking more intensively. Similarly, older men with formal education are found to have a higher likelihood to greater quantities of cigarette.

Compared to male adults affiliated with other religions, those affiliated with Islam and Christianity are found to be less likely to smoke cigarette. This finding is corroborated by Brown et al. [34] who reported an inverse association between smoking among middle-aged men and religious attendance in the United States. The rationale behind this could be the fact that religious men, particularly those of the Islamic and Christian faith relatively frown more on smoking. Notwithstanding, the Christian and Muslim men who smoked were found to smoke more intensively (quantities) than those who belonged to other beliefs. The intensity of smoking among Christian and Moslem men who smoke may be explained by addiction.

Wealthier men are found to have a lower probability to smoke cigarette. Also, among the male adults who smoked, those who lived in the economically disadvantaged and deprived northern regions of Ghana were found to have a higher likelihood to consume greater quantities of cigarette than those who lived in the southern regions of Ghana. This finding is corroborated by Turrell et al. [22] who reported that that disadvantaged neighbourhoods had a lower probability to quit smoking than relatively advantaged neighbourhoods. Therefore, improving poor communities with the objective of curbing smoking is a positive policy direction. Though not significant, the media was found to play an important role in reducing cigarette consumption and its intensity among male adults in Ghana. It signals that when properly harnessed the media is an effective tool for educating the public about the harmful effects of cigarette consumption.

Although the data employed for this study is nationally representative, there are few limitations worth noting. For instance, the sample used in examining the intensity of cigarette consumption is relatively small. It is probable that some smokers did not confess the habit owing to its associated stigma. Nevertheless, since sample weights were applied, the sample is nationally representative and the findings provide some empirical basis for public health policies in Ghana. For more effective policies grounded on this research, additional studies, especially those that use mixed methods- both quantitative and qualitative- are recommended to provide insight on smoking beyond the empirical estimations.

## Conclusions

This paper utilized a nationally representative dataset to explore the factors that affect cigarette consumption as well as smoking intensity among male adults in Ghana.

The study finds that men in lower socioeconomic and demographic category (aged, poor, and low education) have a higher likelihood to smoke. Religion and region of residence are also reported to affect cigarette consumption decision. Furthermore, we find that among the men who smoke, those between the ages of 44 and 60 years and have attained approximately primary education have a higher likelihood to smoke greater quantities of cigarette daily. Also, the smokers who reside in the Upper East and Upper West regions are reported to smoke more intensively than their counterparts in the Greater Accra region. The sampled men who reported smoking were found to smoke as many as about 6 sticks of cigarettes daily on average.

The study’s findings have public health policy implications especially considering the negative impact of second-hand smoking. Imperative public health policies are therefore required. For instance, given the important role played by the media and formal education in discouraging smoking behaviour, these media could be used more intensively in disseminating relevant information on the dangers of smoking. Also, formal education up to at least the secondary level is non-negotiable in combating the dangers of primary and secondary smoking.

## Abbreviations

DHS: Demographic health survey
GDHS: Ghana demographic health survey
GSS: Ghana statistical service
GYTS: Global youth tobacco survey
WHO: World Health Organization

## Footnotes

1 This includes people age 15 years and over who smoke any form of tobacco, including cigarette, cigars, pipes or any other tobacco products.

2 While the study by [1] was carried out in the Greater Accra region (specifically in Accra) by selecting workers from seven (7) ministries, that by [14] was carried out in the Ashanti region.

3 i.e. men who are between 15 and 59 years old

4 The mean of the count variable (smoking intensity) is smaller than its variance.

5 That is, equality in mean and variance.

6 Representing 4.78%.

7 Representing 0.07%.

8 Drawn from the estimated coefficient of the column labelled (4), $\frac{\partial {Smoking\ intensity}_i}{\partial Years\ {of\ Education}_i}=-0.0662083+0.0120334 Years\ {of\ Education}_i=0.$ Thus, *Years of Education*_*i*_ = 5.502 ≈ 6. The second order minimization condition is satisfied.
